# Investigating the Level of Awareness Among the General Population About Various Dental Specialties in Ghaziabad, Uttar Pradesh, India

**DOI:** 10.7759/cureus.85888

**Published:** 2025-06-12

**Authors:** Mukund Sharma, Vikas Singh, Pradeep Tangade, Ankita Jain, Shobhit Goyal, Twinkle Sarawat

**Affiliations:** 1 Public Health Dentistry, Teerthanker Mahaveer Dental College and Research Centre, Moradabad, IND; 2 Oral and Maxillofacial Pathology and Oral Microbiology, Divya Jyoti College of Dental Sciences and Research, Modinagar, IND; 3 Dentistry, Dhanwantri Health and Dental Care Implant Centre, Ghaziabad, IND

**Keywords:** awareness, community survey, dental specialties, education level, ghaziabad, oral health, public health dentistry

## Abstract

Background

The basic knowledge people have about different specialties of dental medicine falls short of what is necessary to achieve complete well-being. The public's insufficient knowledge and misconceptions about specific dental specializations often prevent individuals from seeking appropriate professional treatment. This research evaluated components of dental care awareness among residents of Ghaziabad, Uttar Pradesh, through a population-based survey that explored the relationships between awareness levels and factors such as educational qualification, gender, residential setting, and dental care history.

Materials and methods

Researchers conducted a survey involving 300 participants residing in various areas of Ghaziabad, including rural, suburban, and urban regions. A pre-validated structured questionnaire was used to collect information on demographics, dental treatment history, and recognition of eight dental specialties. Statistical analysis was performed using SPSS v23 to examine the relationships between independent variables and awareness levels using Chi-square tests.

Results

The survey participants were predominantly literate, with 160 (52.8%) identifying as male. Teenagers studying in Ghaziabad demonstrated better recognition of Conservative Dentistry and Endodontics (144; 47.5%), Orthodontics (128; 42.2%), and Oral Surgery (105; 34.3%) compared to other specialties like Public Health Dentistry (77; 25.4%) and Periodontology (88; 29.0%). Gender (p = 0.002), education level (p < 0.001), and number of dental visits (p < 0.001) were significantly associated with awareness levels, whereas type of residence (p = 0.476) showed no significant relationship. Treatment from MDS specialists was received by 145 (48.5%) participants, while general dentists treated 117 (38.9%) of the sample. Previous dental visits were reported by 188 (62.7%) of respondents, most of whom preferred private clinics for their treatment.

Conclusion

Despite relatively high literacy levels, the population of Ghaziabad demonstrates poor awareness of dental specialties. Individuals with higher educational attainment and frequent dental visits exhibited better understanding of specialized dental services. There is a pressing need for targeted educational programs and public health initiatives to improve awareness of dental specialties and enhance access to appropriate dental care.

## Introduction

Oral health significantly impacts overall systemic health. Despite advancements in dental care, including specialized fields such as prosthodontics, orthodontics, and oral surgery, many individuals remain unaware of these services. This lack of awareness contributes to misconceptions about dental care and underutilization of specialized services [1]. Existing literature indicates that people still expect to lose their teeth with age and believe that nothing can be done to prevent it [2]. Furthermore, research has shown that the majority of individuals who seek dental care do so only when symptoms arise [3]. Factors such as fear and anxiety, the availability of home remedies, and socioeconomic conditions significantly influence individuals' oral health behaviors and dental visitation patterns. However, a person’s attitude toward oral health and dental professionals remains a somewhat modifiable barrier to seeking care [4]. Educational background, cultural diversity, and social stratification also contribute significantly to variations in attitudes toward oral health [5]. Currently, all scientific disciplines, including dental health, are rapidly evolving. Moreover, research has highlighted the growing necessity of dental specialization [6].

While the average person can typically distinguish between a cardiologist and a neurologist, they often cannot differentiate between various dental specialties. Despite progress in dental specialization, many patients remain unaware of the specific qualifications of their treating dentist [7]. In recent years, numerous innovations have emerged to enhance dentition both cosmetically and functionally, including implant technology, laser applications, geriatric dental care, and preventive oral health services. Nonetheless, awareness of dental specialties remains limited. In contrast, it is also important to recognize that general dentists may be reluctant to refer patients to specialists due to concerns about losing clientele [8]. This study aims to assess the level of awareness among residents of Ghaziabad regarding various dental specialties and to test the hypothesis that awareness significantly varies by education level, residential area (urban, suburban, rural), and frequency of dental visits.

## Materials and methods

The researchers conducted this descriptive cross-sectional study in Ghaziabad, Uttar Pradesh, to evaluate public understanding of dental specialties. The Institutional Ethical Committee of Teerthanker Mahaveer Dental College and Research Centre approved the ethical clearance for this research (approval number: TMDCRC/IEC/SS/25-26/PUBHEALDENT 03). The study was conducted from April 20, 2025 to April 27, 2025.

The sample size was calculated using a convenience sampling method with the formula: n = Z² × p × (1-p) / d²

where Z = 1.96 for a 95% confidence level, p = anticipated proportion of awareness (0.5 or 50%) [2], and d = margin of error. Using this formula, the minimum required sample size was 267. To account for potential non-responses or incomplete data, the final sample size was increased to 300 participants. The calculation was performed using G*Power software.

A total of 300 subjects participated from various rural and urban locations across Ghaziabad to ensure balanced representation. Participants were informed about the study objectives and provided consent before data collection began. Adult residents of Ghaziabad aged 18 and above were eligible to participate if they voluntarily chose to take part in the study.

A structured questionnaire with strong face validity (score = 1) was developed in English, validated, and pre-tested. It was then translated into Hindi to facilitate better understanding among non-English-speaking participants (Appendix 1). Content validity was assessed by a panel of five dental experts who reviewed the questionnaire for relevance, clarity, and comprehensiveness. The final version was prepared after reaching a consensus and pilot-tested on a small sample of the target population. The questionnaire demonstrated a Cronbach’s alpha of 0.82, indicating good internal consistency. The survey contained three segments, beginning with demographic inquiries about age, gender, educational level, and residential status. This was followed by questions related to dental appointments, including visit frequency, types of services availed, and preferred healthcare centers. The final segment focused on awareness of recognized dental specialties, including Oral Medicine and Radiology, along with six other major specialties: Conservative Dentistry and Endodontics, Orthodontics, Prosthodontics, Public Health Dentistry, Pedodontics and Periodontology, and Oral and Maxillofacial Surgery.

Data collection was carried out through face-to-face interviews conducted by a team of two trained investigators, both with a background in dentistry. The investigators underwent a structured training session prior to data collection, focusing on interview techniques, ethical guidelines, and the standardization of questionnaire administration to ensure data reliability and minimize interviewer bias. Interviews were conducted directly with participants who attended dental outreach programs and visited both private and public healthcare facilities in Ghaziabad.

The authors processed the data using IBM SPSS Version 23, while Microsoft Excel was used for data compilation. Demographic information and response data were analyzed using descriptive statistics, with results presented as frequencies and percentages. To examine correlations between awareness of dental specialties and independent variables, including gender, literacy status, education level, place of residence, and number of dental visits, the study employed Chi-square tests. A significance level of 0.05 was used as the statistical threshold. The analysis provided insights into public awareness of dental specialties and the factors influencing this knowledge.

## Results

A total of 300 participants from various demographic areas of Ghaziabad took part in this study. Table 1 shows that 52.8% of the participants identified as male, while 46.2% identified as female.

**Table 1:** Percentage distribution of subjects by gender, literacy status, education level, and place of residence.

Parameter	Category	Frequency (%)
Gender	Male	160 (52.8)
	Female	140 (46.7)
Literacy Status	Literate	284 (94.7)
	Illiterate	16 (5.3)
Education Level	Primary School	51 (17.0)
	Middle School	60 (20.0)
	High School	56 (18.7)
	Pre-University	64 (21.3)
	Graduation	69 (23.0)
Residence	Rural	87 (29.0)
	Suburban	85 (28.3)
	Urban	128 (42.7)

Nearly all participants, 284 (93.7%), demonstrated literacy, while only 16 (5.3%) were classified as illiterate. Graduation-level education was the most common level of attainment, reported by 69 (22.8%) participants, followed by pre-university education at 64 (21.1%). The distribution among other education levels included high school (56; 18.5%), middle school (60; 19.8%), and primary education (51; 16.8%). Most participants were from urban areas (128; 42.2%), followed by rural (87; 28.7%) and suburban (85; 28.1%) regions.

People interpreting test results from a gender perspective discovered that males exhibited superior dental specialty awareness compared to females (χ²=12.103, p=0.002). However, the level of awareness between literate and illiterate participants was not statistically significant (χ² = 2.076, p = 0.354). A strong association was observed between education level and knowledge of dental specialties, with participants having higher educational qualifications showing better awareness (χ² = 35.596, p < 0.001). Interestingly, while the association was significant, the highest levels of awareness were not always among the most highly educated individuals, suggesting that factors beyond formal education may influence awareness, as shown in Table 2.

**Table 2:** Percentage of subjects by dental visit frequency and awareness of dental specialties.

Parameter	Category	Awareness: Yes	No	Can't Say	Chi-square (p-value)
Gender	Male	62	89	9	χ² = 12.103, p = 0.002
	Female	29	104	7	
Literacy Status	Literate	84	184	16	χ² = 2.076, p = 0.354
	Illiterate	7	9	0	
Education Level	Primary School	15	36	0	χ² = 35.596, p < 0.001
	Middle School	23	31	6	
	High School	17	29	10	
	Pre-University	19	45	0	
	Graduation	17	52	0	
Place of Residence	Rural	29	52	6	χ² = 3.518, p = 0.476
	Suburban	28	55	2	
	Urban	34	86	8	
Number of Dental Visits	1-2	77	11	3	χ² = 33.952, p < 0.001
	3-4	105	73	15	
	>5	16	0	0	

The number of dental visits significantly influenced awareness of dental specialties. Participants who had visited dental clinics more than five times showed considerably higher awareness compared to those with fewer visits. This association was also highly significant (χ² = 33.952, p < 0.001). The most recognized specialty among respondents was Conservative Dentistry and Endodontics (144; 47.5%), followed by Orthodontics and Dentofacial Orthopedics (128; 42.2%), and Oral and Maxillofacial Surgery (104; 34.3%). As shown in Table 3, Public Health Dentistry (77; 25.4%) and Periodontology (90; 29.0%) were among the least recognized specialties.

**Table 3:** Awareness levels (%) of the study population regarding different dental specialties.

Dental Specialty	Awareness (%)
Oral Medicine and Radiology	101 (33.7)
Conservative Dentistry and Endodontics	144 (47.5)
Orthodontics and Dentofacial Orthopedics	128 (42.2)
Prosthodontics and Crown and Bridge	105 (34.7)
Public Health Dentistry	77 (25.4)
Pedodontics and Preventive Dentistry	90 (29.7)
Periodontology	88 (29.0)
Oral and Maxillofacial Surgery	104 (34.3)

Public awareness was primarily focused on restorative and corrective specialties, rather than preventive and community-oriented dental practices. In terms of treatment, dental specialists (MDS) provided services to 145 (48.5%) of the participants, while general dentists (BDS) treated 117 (38.9%), and 35 (11.6%) participants were unsure of their provider’s qualifications. A majority of participants (188; 62.7%) had visited a dental clinic, with 166 (55.3%) preferring private practitioners for care. Among the total, 89 (29.7%) had visited dental colleges, 55 (18.2%) attended private hospitals, and 47 (15.8%) received care in government hospitals. The most commonly received procedures were fillings and extractions, while some participants reported undergoing orthodontic treatments. The least reported procedures were periodontal and preventive care, reflecting a lack of awareness and limited utilization of holistic dental services.

## Discussion

The study results indicate that residents of Ghaziabad, Uttar Pradesh, possess a notable level of awareness about different dental specialties. However, a high literacy rate, 284 (93.7%), along with the presence of graduates and high school completers among participants, did not correlate with satisfactory awareness of dental specialty branches. These results suggest that while general education levels have improved, oral health literacy, particularly regarding specialty care, has not kept pace. Research findings also showed that participants from urban areas made up over half of the sample, likely due to the assumption that urban regions offer better access to healthcare facilities and information. Nonetheless, these participants demonstrated limited awareness of dental specialties such as periodontology, pedodontics, and public health dentistry. Most people perceived dentists primarily as providers of basic procedures like fillings and extractions, which explains why awareness was highest for conservative dentistry and endodontics. Advanced dental care practices remain largely unknown to the public due to a lack of awareness.

The belief that general dentists provide all types of dental treatment persists among many patients, who often do not seek care from specialists, even when needed, due to misconceptions about specialist roles. These outdated perceptions, rooted in past generations, continue despite improvements in general education. Public awareness of specialized dental care remains low, as shown in a study conducted by Umesi-Koleoso DC and Ayanbadejo PO [9]. This issue is further compounded by the limited awareness exhibited by medical professionals and students. Research conducted by Adeghe H et al., Shenoy P et al., and Nagrik AP et al. confirms that medical students often have insufficient understanding of dental specialties, largely because their information sources are informal [10-12]. Inadequate exposure to dental specialties during medical training hampers interdisciplinary collaboration and affects both patient referral behavior and clinical outcomes. In our study, statistical associations between education level and dental awareness, as well as between dental visits and awareness, failed to demonstrate a broad-based understanding or consistent use of specialty dental care [13].

Participants who visited the dentist more frequently exhibited significantly better awareness of dental specialties. This finding is supported by the work of Jamieson LM and Thomson M, who noted that patients familiar with dental services are more likely to undergo treatment and follow professional recommendations [14, 15]. Our findings are also in line with Shenoy R et al., showing that 188 (62.7%) of participants had prior experience with dental treatments [16], a proportion comparable to the 68% reported by Shenoy R et al. These statistics highlight the widespread avoidance of dental care and the lack of awareness among both recipients and non-recipients regarding their dentist’s qualifications or area of expertise. As noted by Gummaluri SS et al. [15], public understanding of specialized dental fields remains inadequate due to the lack of sustained educational initiatives. Progress in oral healthcare remains constrained by cultural beliefs, social stigmas, insufficient structured awareness programs, and the limited integration of oral health into primary healthcare systems.

Multiple coordinated strategies need to be implemented to address the existing shortcomings. Public health initiatives should establish community-based programs that educate people about dental specializations and their importance. These campaigns must be culturally sensitive and accessible to individuals across various literacy levels. Integrating information about dental specialties into school curricula can foster early awareness of oral health and help prevent dental issues from a young age. Stronger collaboration between general practitioners, medical doctors, and dental specialists is essential to ensure comprehensive patient care and improve referral processes. Public trust in specialist dental care can be strengthened through cross-disciplinary communication, ultimately leading to better treatment outcomes. The research offers useful findings, but its broad adoption could be restricted because the sample consisted of residents from Ghaziabad. The national patterns in dental specialty awareness require additional research that combines multiple regions using extensive sample collections to create specific intervention strategies.

The study limitations are that the sample was limited to residents of Ghaziabad, which may not accurately represent the awareness levels or healthcare access in other parts of India. As a result, the findings may have limited generalizability to the national population. Cultural, educational, and socioeconomic differences across regions could influence public awareness and perceptions of dental specialties, and these factors were not fully explored in the study. Additionally, to ensure clear communication through the use of trained investigators and a translated questionnaire, respondent bias may have occurred during face-to-face interviews, especially among participants with lower literacy levels.

## Conclusions

A study emphasizes the necessity of targeted dental awareness efforts to raise public understanding of dental specialty disciplines. Improved utilization of specialized services can enhance oral health outcomes across Ghaziabad. These programs should incorporate schools, community involvement, and media-based education to combat misconceptions about dental specialties and encourage early treatment-seeking behavior. Increasing accurate dental awareness among the population will help bridge the gap between basic and specialized dental services while fostering an informed and health-oriented society.

## Appendices

Appendix 1

Structured questionnaire for assessing public awareness of dental specialties

Demographic Details

Name:

_____________________________

Age:

_____________________________

Sex:

_____________________________

Q1. Literacy Status

☐ Literate ☐ Illiterate

Q2. Education Level (if literate)

☐ Primary School ☐ Middle School ☐ High School

☐ Post High School/Pre-University ☐ Graduation

| Q3. Area of Residence | ☐ Rural Area ☐ Sub-Urban Area ☐ Urban Area |

| Q4. Previous Dental Treatment | ☐ Yes ☐ No |

Q5. (If Yes to Q4) Treatment Details

5a. Number of Dental Appointments

_____________________________

5b. Type of Dental Set-up

☐ Private Clinic ☐ Dental College ☐ Private Hospital ☐ Govt. Hospital

5c. Type of Treatment Rendered

☐ Filling ☐ Tooth Removal ☐ Teeth Straightening/Wire Treatment

☐ Teeth Cleaning/Gum Treatment ☐ Teeth Replacement ☐ Other: ______

5d. Qualification of Treating Doctor

☐ General Dentist (BDS) ☐ Specialist (MDS) ☐ Can’t Say

| Q6. Awareness of Dental Specialties (8) | ☐ Yes ☐ No ☐ Can’t Say |

Q7. Awareness of Specialties for the Following Treatments

Response

7a. Dental check-up’s/Dental X-ray’s

☐ Yes ☐ No ☐ Can’t Say

7b. Filling of decayed/broken teeth

☐ Yes ☐ No ☐ Can’t Say

7c. Straightening of crooked teeth (wires/braces)

☐ Yes ☐ No ☐ Can’t Say

7d. False teeth sets/dentures, caps/bridges

☐ Yes ☐ No ☐ Can’t Say

7e. Dental camp organization

☐ Yes ☐ No ☐ Can’t Say

7f. Dental treatment of children

☐ Yes ☐ No ☐ Can’t Say

7g. Gum treatment and teeth cleaning

☐ Yes ☐ No ☐ Can’t Say

7h. Tooth removal and jaw fracture treatment

☐ Yes ☐ No ☐ Can’t Say

| Q8. Is it important for patients to know about dental specialties? | ☐ Yes ☐ No ☐ Can’t Say |
